# Impact of access to childhood health services on healthy life expectancy of the older population

**DOI:** 10.3389/fpubh.2023.1234880

**Published:** 2023-09-19

**Authors:** Chen Liu, Xiaochun Hou, Qiong Wang, Xinrui Xu, Bingyi Wu, Jun Liu

**Affiliations:** Department of Management, Weifang Medical University, Weifang, Shandong, China

**Keywords:** life course theory, health services, healthy life expectancy, probability of transition risk, multi-state life table

## Abstract

**Background:**

Life course theory provides new perspectives on the impact of early experiences on health in old age, where unfortunate childhood experiences can alter an individual's health trajectory. This study aims to calculate the healthy life expectancy of the older population in China under different childhood experiences, and to explore the influence of childhood medical and health services on the health level of older adults.

**Methods:**

Differences in healthy life expectancy of the older population under different childhood experiences were analyzed using the multi-state life table method to calculate the healthy life expectancy by sex and place of birth, based on the cohort data of Chinese Longitudinal Healthy Longevity Survey (CLHLS) from 2008 to 2018.

**Results:**

The probability of the transition risk from health to non-health gradually increased with age, while the probability of the transition risk from non-health to health decreased with age; In both urban and rural areas, on the probability of the transition risk from health to non-health, the older adults who were able to receive timely medical and health services in childhood were lower than those who failed to receive medical services in time (*Z* = −5.833, *P* < 0.05), but the probability of the transition risk from non-health to health was the opposite (*Z* = −5.334, *P* < 0.05); The probability of the transition risk from health to death is also higher in older adults who were unable to receive timely medical care in childhood (*Z* = −5.88, *P* < 0.05); The healthy life expectancy and its proportion in the remaining life expectancy of older people who received medical and health services in time during childhood were significantly higher than those of their peers (*Z* = −5.88, *P* < 0.05).

**Conclusions:**

The lack of medical services in childhood has a negative effect on the health of older adults. The healthy life expectancy and its proportion of remaining life expectancy were higher for rural older adults than for urban older adults under the same health care conditions in childhood; the health benefits of good access to health care environment or conditions in childhood were greater for rural older females.

## 1. Introduction

China is the country with the largest older population in the world currently, with both the large absolute number of older people, and the rapid aging process, so the health problems of older adults are becoming increasingly prominent (1). The health of older adults is not only related to the quality of life of individuals and the burden of family life, but also related to the wellbeing of the whole society (2). As a special group with increasing health vulnerability and disease risks, older adults are facing increasingly serious health inequalities among people with different socioeconomic status (3).

Life course theory is an important theoretical perspective for the study of older people's health (4). According to life course theory and the Developmental Origin Hypothesis of Health and Disease (DOHaD), childhood is an important stage in an individual's development, so that experiencing adverse living conditions or events during this period can adversely affect later health outcomes (5), while health inequalities are rooted in inequalities experienced during fetal period, childhood, and adolescence (6). According to the integrative health medicine model, medical care is one of the four major influencing factors of health throughout the life cycle. However, in terms of health care, it is believed that the highest manifestation of fairness is that everyone can have fair and equal access to medical service resources (7). During the political turmoil and lack of materials in China from 1930's to the early years of the People's Republic of China, those born and raised during this period were likely to face more unequal access to health care. From the perspective of life course theory, medical services in childhood play an important role to the health of this age group. Good health resources are conducive to promoting and ensuring the health level of childhood, and the advantages or disadvantages of childhood health affect the health status in the middle and later stages of life course through cumulative effects, among which medical and health services are important pathways in the mechanism of action (8). The impact of health services on the health of older adults in this mechanism of action can be reflected in the differences in healthy life expectancy among populations.

Healthy life expectancy is a powerful tool for measuring the overall health status of the population and assessing health needs and health burdens, it can examine the distribution of population health status (9). This indicator contains information that reflects both mortality and prevalence, which can effectively reflect the life quality of the population (10). The differences in healthy life expectancy among the older population can be explained by different factors, including the level of economic development, social wealth distribution system, and medical services (11). Therefore, exploring healthy life expectancy is conducive to better understanding and grasping the health level and changing trend of older adults in the context of population aging, so it serves as an important basis for actively responding to population aging. Most of the existing literature on the impact of childhood experiences on health in old age focuses on the family environment, socioeconomic status, social interactions, and social environment during childhood (5, 12). However, in studies about older Chinese people, the importance of health care services in the process of health level changes throughout the life cycle of individuals is not explored. Also, the indicators used to measure health level are mostly single measures of physical or psychological aspects of old age, which do not comprehensively reflect the quality of life of the older population (13–16). Additionally, cross-sectional data does not reflect the overall development trend of the health of the older population, so it is insufficient to have an overall understanding of the mortality and health level of the older population in China.

To this end, this study intends to measure the healthy life expectancy of the older population in China under different childhood experiences using CLHLS cohort data from 2008 to 2018, so as to explore the influence of childhood medical and health services on the health level of older adults. Based on the life course theory, the study compares the differences in healthy life expectancy and related indicators among the older population aged 65 and above under different childhood experiences. The goal is to fully explore the problem of health inequality in older adults, enrich the research on the relationship between childhood adversity and older people's health under life course theory, and then provide an empirical basis for the formulation of personalized older people's health promotion strategies.

## 2. Research sources and methods

### 2.1. Research sources

The 2008–2018 data of the Chinese Longitudinal Healthy Longevity Survey (CLHLS) are used in this study, with a multi-stage sampling method, covering 23 provinces including autonomous regions and municipalities directly under the central government in China. The research subjects are older adults aged 65–105, and the 2008 survey is used as the baseline data for the study, excluding the newly included samples in the subsequent three surveys. After weighted adjustment, the final sample size is 14,261 in 2008, 10,599 in 2011, 8,596 in 2014 and 5,159 in 2018.

### 2.2. Variable settings

The core independent variable of this study, “accessibility of childhood health services,” is a dichotomous variable. According to the CLHLS questionnaire item, “Could you get adequate medical service when you were sick in childhood?,” the answer “yes” is defined as “accessible to childhood health services” and assigned a value of “1,” while the answer “no” is defined as “inaccessible to childhood health services” and assigned a value of “0.”

The dependent variable is healthy life expectancy, this paper uses self-rated health as health indicator to calculate healthy life expectancy. For the CLHLS survey item “How do you rate your health at present?” The answers “very good,” “good,” “not bad” are defined as “healthy,” and the value is assigned as “1”; the answers “bad” and “very bad” are defined as “non-health” and assigned a value of “2.” According to IMaCH software, when older adults subjects die or fail to follow-up, the values are assigned as “3” and “−1” respectively.

The covariates are gender, place of birth, education level and economic level. In gender variables, the female assignment is “0” and the male assignment is “1”; Since the study design focuses on the impact of access to health services in childhood on the health of the older adults, the place of birth of the survey participants was selected as the urban-rural variable for heterogeneity analysis. For the question “Was your place of birth in an urban or a rural area (at time of birth)?,” the answer “urban” is assigned as “1” and “rural” as “0.” Defined as “non-literate” and assigned a value of “1” if the respondent has had any education, otherwise assigned a value of “0.” We use respondents' annual household income to measure their economic level.

### 2.3. Multi-state life table

The study used IMaH software to measure the healthy life expectancy of older adults by multi-state life table method. The status of the surveyed older adults is divided into three types: health, non-health, and death; The two survival states of “health” and “non-health” can be mutually transferred, which are “non-absorbing states” in the model while “death” is the “absorbed state,” so it is irreversible between “health” and “death,” as well as “non-health” and “death.”

**Figure 1:**
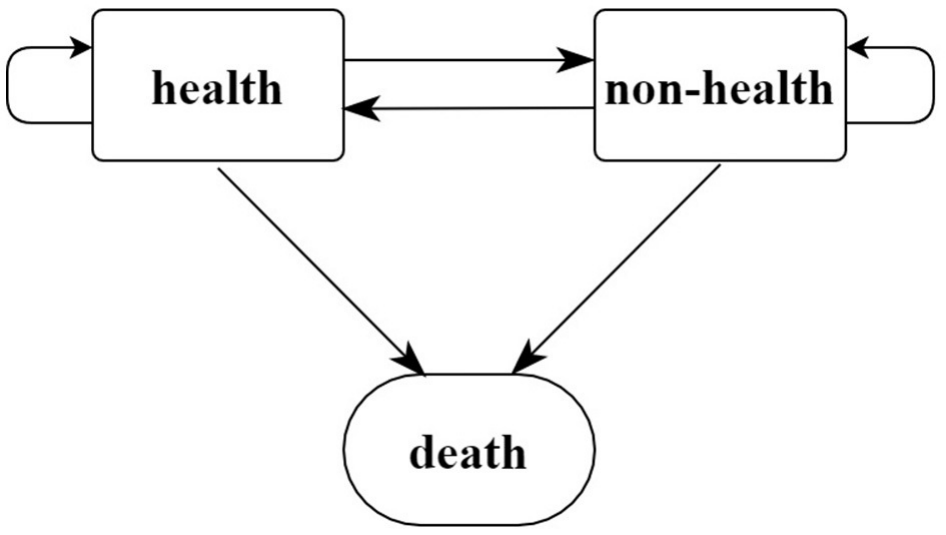
States and transition paths in a multi-state life table.

The specific conversion path is shown in Figure 1.

Assuming that the transition between healthy states follows the first-order Markov chain setting, the individual's state at age *x* is *j*, and the probability of the state at age *x*+*h* is *k* ${}_{h}p^{jk}=Pr\;\left(X\;\left(x+h\right)=k|X\;\left(x\right)=j\right)$. Let $L_{t_{1}-t_{2}}^{\left(i\right)}$ represent the probability${}_{d1}p_{x1}^{jk}$ of the transition of an individual from state *j* to state *k* between the two surveys *t*_1_ and *t*_2_, and if two follow-up surveys are performed, the probability of the individual being *i* state transition is $L^{\;(i)}=\left({}_{d1}p_{x1}^{jk}\right)*\left({}_{d2}p_{x2}^{k1}\right)$; If the state at the beginning of the period is *i* and the state at the end of the period is 1 (healthy) or 2 (non-health), then the probability of health and the probability of non-health are ${}_{t}w^{i1}(x)=\frac{{}_{t}p_{x-t}^{i1}}{{}_{t}p_{x-t}^{i1}{+}_{t}p_{x-t}^{i2}}$ and ${}_{t}w^{i2}(x)=\frac{{}_{t}p_{x-t}^{i2}}{{}_{t}p_{x-t}^{i1}{+}_{t}p_{x-t}^{i2}}$, respectively; The stable prevalence of individual outcomes *j* was ${}_{y}p_{x}^{j}(\theta )={}_{y}p_{x}^{1j}(\theta )+w^{2}(x,\theta ){(}_{y}p_{x}^{2j}(\theta ){-}_{y}p_{x}^{1j}(\theta ))$; then the healthy life expectancy in the age range (*x, x*+*y*) is ${}_{y}e_{x}^{ij}=\sum_{u=1}^{y}{}_{u}p_{x}^{ij}$, while the life expectancy in different initial states is $e_{x}^{i.}=e_{x}^{i1}+e_{x}^{i2}$; the overall life expectancy at age X is $e_{x}^{..}=e_{x}^{.1}+e_{x}^{.2}$ (17).

### 2.4. Statistical analysis

First, the survey data were weighted using the weight coefficients provided by the CLHLS project team, and the weighted data were compared to the sixth census data of China from the age and sex, making the study sample more representative. Second, Stata 14.0 software was used to describe the frequency of different health statuses of the older adults. It analyzed the distribution of health status by age, gender, place of birth, education level, income level, and access to childhood health services. Third, chi-square test was used to explore the differences in the socioeconomic status of older people with or without health services in childhood, taking α = 0.05 as the inspection standard. Finally, by IMaCh software, the multi-state life table method was used to measure the life expectancy and healthy life expectancy of the older people who have timely health services or not in childhood. Fourth, statistical differences between healthy life expectancy were analyzed by rank tests.

## 3. Outcome

### 3.1. Data quality assessment

**Figure 2:**
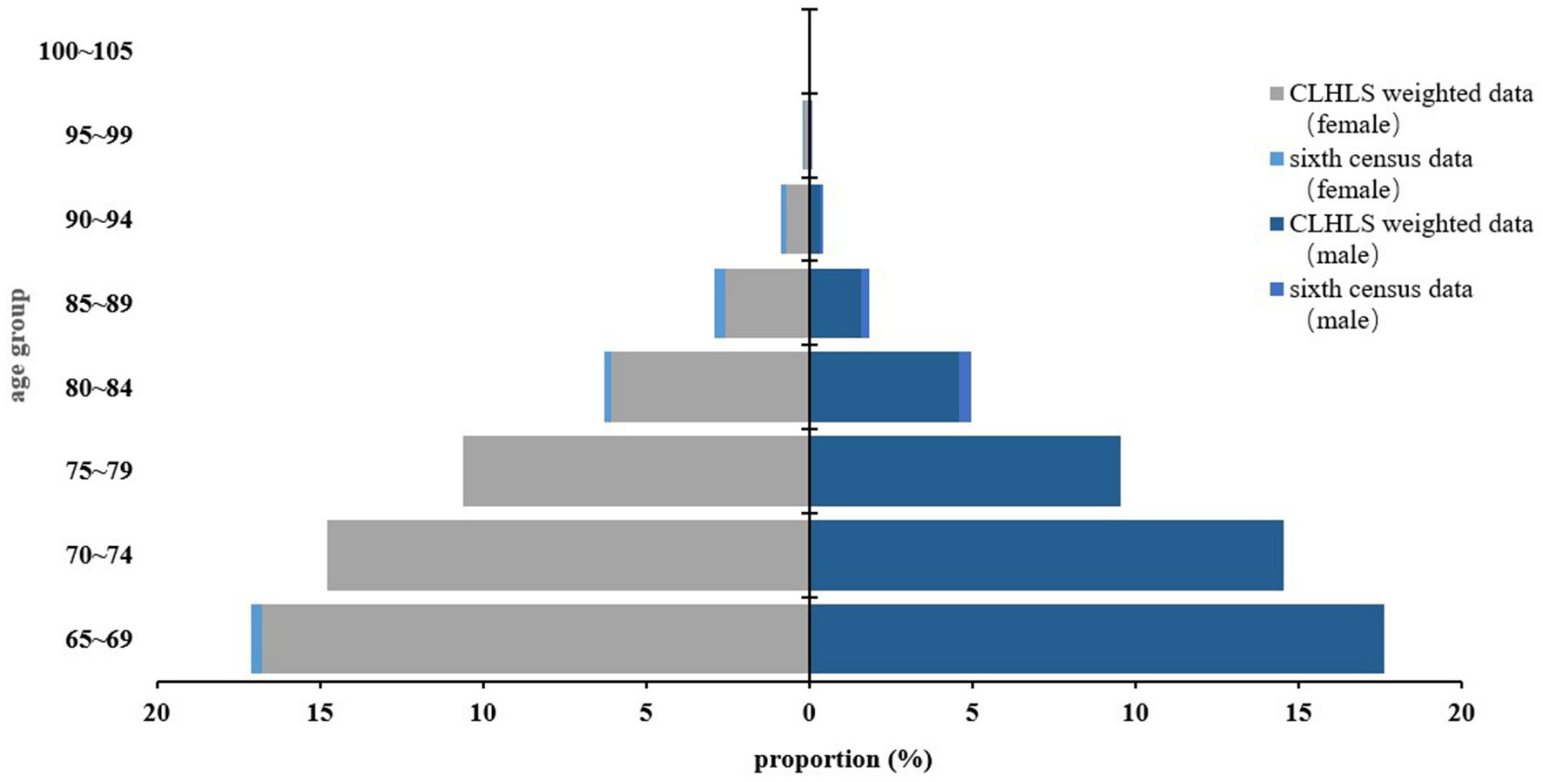
Comparison between the sixth census data of China and weighted survey data by sex–age structure.

Taking the sex–age structure of older adults over 65 years in the sixth census in China as a reference, the data after weight adjustment of the cohort in the CLHLS database from 2008 to 2018 were compared. The results showed that the weighted adjusted data fitted well with the sixth census data of China (Figure 2).

### 3.2. Basic information

Table 1 shows the basic situation of the older adults after weighted adjustment for the four surveys. This study uses cohort data from 2008 to 2018, with 2008 data as the baseline, and the population numbers of the four surveys after weighted adjustment are 14,261, 10,599, 8,596, and 5,159, respectively. In terms of age structure distribution, the group aged from 65 to 69 has the largest proportion, accounting for 34.41%, and over time, the proportion gradually decreased in the total population. As of the 2018 survey, the proportion of older people in this age group is 0. The proportion of older adults aged 100 and above is the smallest, accounting for 0.04%, 0.04%, 0.11%, and 0.15% in the four surveys, respectively. From the perspective of gender, the proportion of female older adults is relatively large, with more than half of the four surveys, accounting for 51.69%, 52.62%, 53.41%, and 56.30% respectively, while the number of male older adults gradually decreases. As far as the childhood location of the older adults is concerned, most of them are concentrated in rural areas, with the proportion of rural areas in the baseline survey period being 84.23%, and by 2018, the proportion was as high as 90.00%, while the proportion of urban areas was relatively small. Most of the older adults did not receive timely medical services in childhood, and the proportion fluctuated in the four surveys, which were 57.72%, 59.04%, 58.71%, and 59.58% respectively. More than half of the older adults have not received any education; the annual household income of the the older adults is around RMB 20,000 to RMB 40,000 per year. From the perspective of the health status of the older adults, the proportion of older people in healthy state in 2008 was the largest, up to 90.58%, while the proportion of unhealthy older adults was < 10%; By 2011, there were 75.89% healthy older adults and 11.53% unhealthy older adults, 4.89% death and 7.68% loss to follow-up respectively; In 2014, the proportion of healthy older adults was 51.18%, and unhealthy older adults 7.69%, and the proportion of death and loss to follow-up was 35.55% and 5.57% respectively. In 2018, the proportion of healthy older adults was 29.51%, and unhealthy older adults 5.86%. The proportion of death and loss to follow-up older adults was 54.33% and 10.30% respectively.

**Table 1:** Basic information of the sample older population.

**Variables**		**2008**		**2011**		**2014**		**2018**	
		***n***	**Proportion (%)**	***n***	**Proportion (%)**	***n***	**Proportion (%)**	***n***	**Proportion (%)**
Age	65–69	4,907	34.41	1,558	14.70	1	0.02	0	0
	70–74	4,181	29.32	3,641	34.35	2,825	32.86	51	0.99
	75–79	2,878	20.18	2,882	27.19	2,839	33.03	2,251	43.63
	80–84	1,519	10.65	1,609	15.18	1,843	21.44	1,632	31.63
	85–89	593	4.16	690	6.51	803	9.34	856	16.60
	90–94	147	1.03	182	1.72	236	2.75	300	5.82
	95–99	30	0.21	32	0.30	40	0.46	61	1.18
	100–105	7	0.04	4	0.04	9	0.11	8	0.15
Gender	Female = 0	7,372	51.69	5,577	52.62	4,591	53.41	2,905	56.30
	Male = 1	6,889	48.31	5,022	47.38	4,005	46.59	2,254	43.70
Place of birth	Rural = 0	12,012	84.23	9,184	86.65	7,475	86.96	4,650	90.13
	Urban = 1	2,249	15.77	1,415	13.35	1,121	13.04	509	9.87
Education level	Literate	8,883	62.29	5,989	56.50	4,595	53.46	2,527	48.99
	Non-literate	5,378	37.71	4,610	43.50	4,001	46.54	2,632	51.01
Economic level	Quantitative variable	22,308 ± 26,076		26,422 ± 15,501		35,608 ± 32,006		43,732 ± 36,248	
Childhood health services	Not accessible =0	8,231	57.72	6258	59.04	5,047	58.71	3,074	59.58
	Accessible = 1	6,030	42.28	4,341	40.96	3,549	41.29	2,085	40.42
Geriatric health conditions	Missing visits = −1	0	0	1,095	7.68	794	5.57	7,748	54.33
	Health = 1	12,918	90.58	10,823	75.89	7,299	51.18	4,208	29.51
	Non-health = 2	1,343	9.42	1,644	11.53	1,097	7.69	836	5.86
	Death = 3	0	0	697	4.89	5,070	35.55	1,469	10.30
N		14,261		10,599		8,596		5,159	

The above numbers for 2008, 2011, 2014 and 2018 are obtained from CLHLS weighted calculations.

### 3.3. Single-factor analysis

The differences in the socioeconomic status of the two groups of older adults were explored. The results showed that there was a statistically significant difference in age, gender, residency, education level, and income level between the groups with or without (*P* < 0.05). Specifically, the main features of the older adults who do not have timely access to health services during childhood are as follows: mainly over 85 years old, female (38.86%), rural (60.37%), lower education level (45.32%). The detailed results are shown in the Table 2.

**Table 2:** Analysis on the differences in social attributes of childhood health services.

**Variable**		**Childhood health services**		**χ^2^**	***P***
		**Not accessible**	**Accessible**		
Age	65–69	715 (5.20%)	534 (3.88%)	132.18	< 0.000
	70–74	793 (5.76%)	556 (4.04%)		
	75–79	783 (5.69%)	451 (3.28%)		
	80–84	1,124 (8.17%)	599 (4.35%)		
	85–89	1,330 (9.66%)	619 (4.50%)		
	90–94	1,632 (11.86%)	817 (5.94%)		
	95–99	851 (6.18%)	431 (3.13%)		
	100–105	1,835 (13.33%)	691 (18.36%)		
Gender	Female	5,348 (38.86 %)	2,464 (17.91%)	54.28	< 0.000
	Male	3,715 (27.00%)	2,234 (16.23%)		
Place of birth	Rural	8,308 (60.37%)	3.687 (26.79%)	481.15	< 0.000
	Urban	755 (5.49%)	1,011 (7.35%)		
Education level	Literate	6,237 (45.32%)	2,335 (16.97%)	481.37	< 0.000
	Non-literate	2,826 (20.54%)	2,363 (17.17%)		

### 3.4. Health transition probability

**Figure 3:**
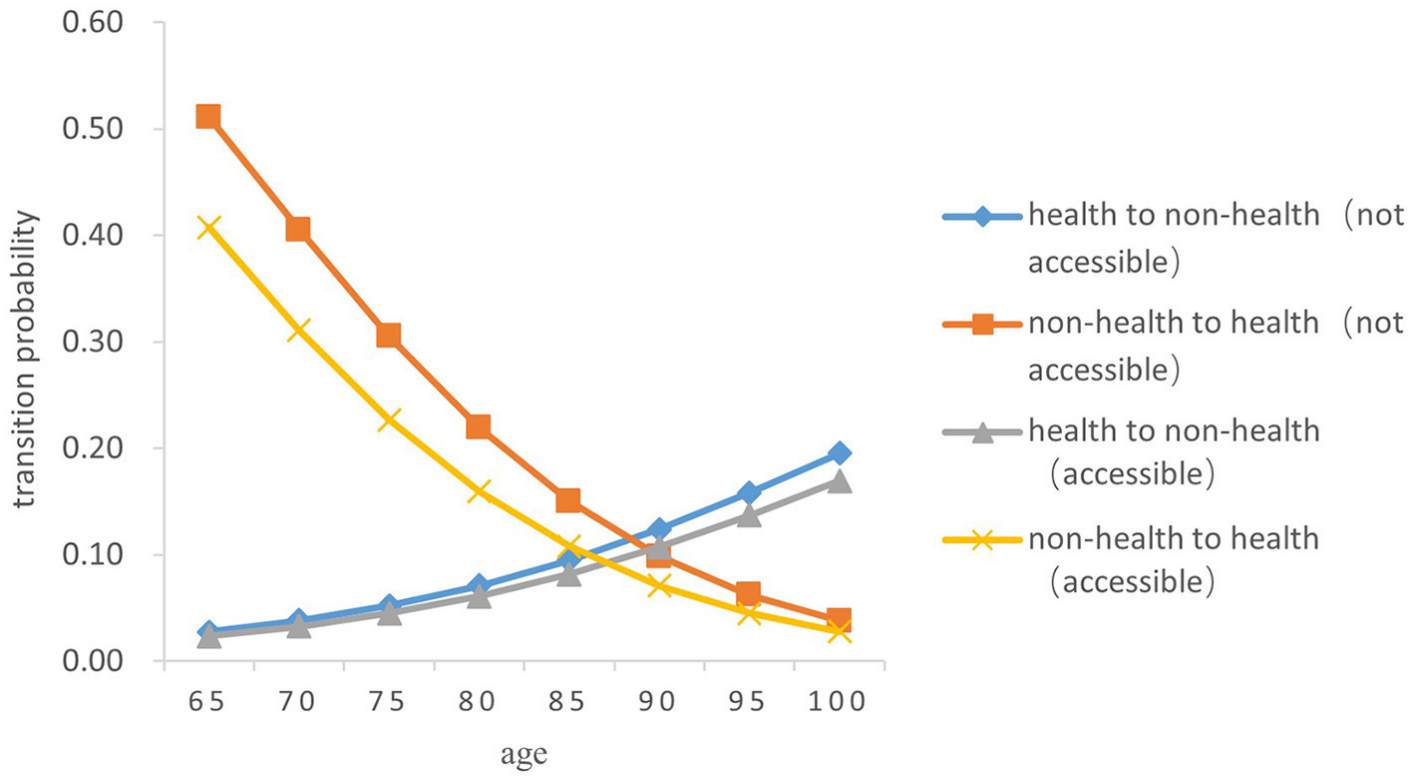
Transition probability curves between health and non-health of rural older female.

**Figure 4:**
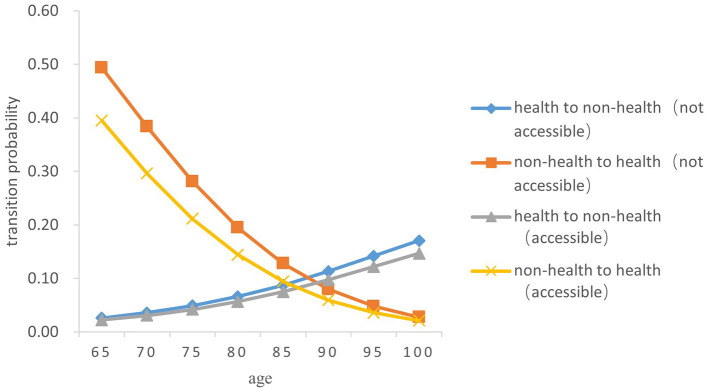
Transition probability curves between health and non-health of rural older male.

**Figure 5:**
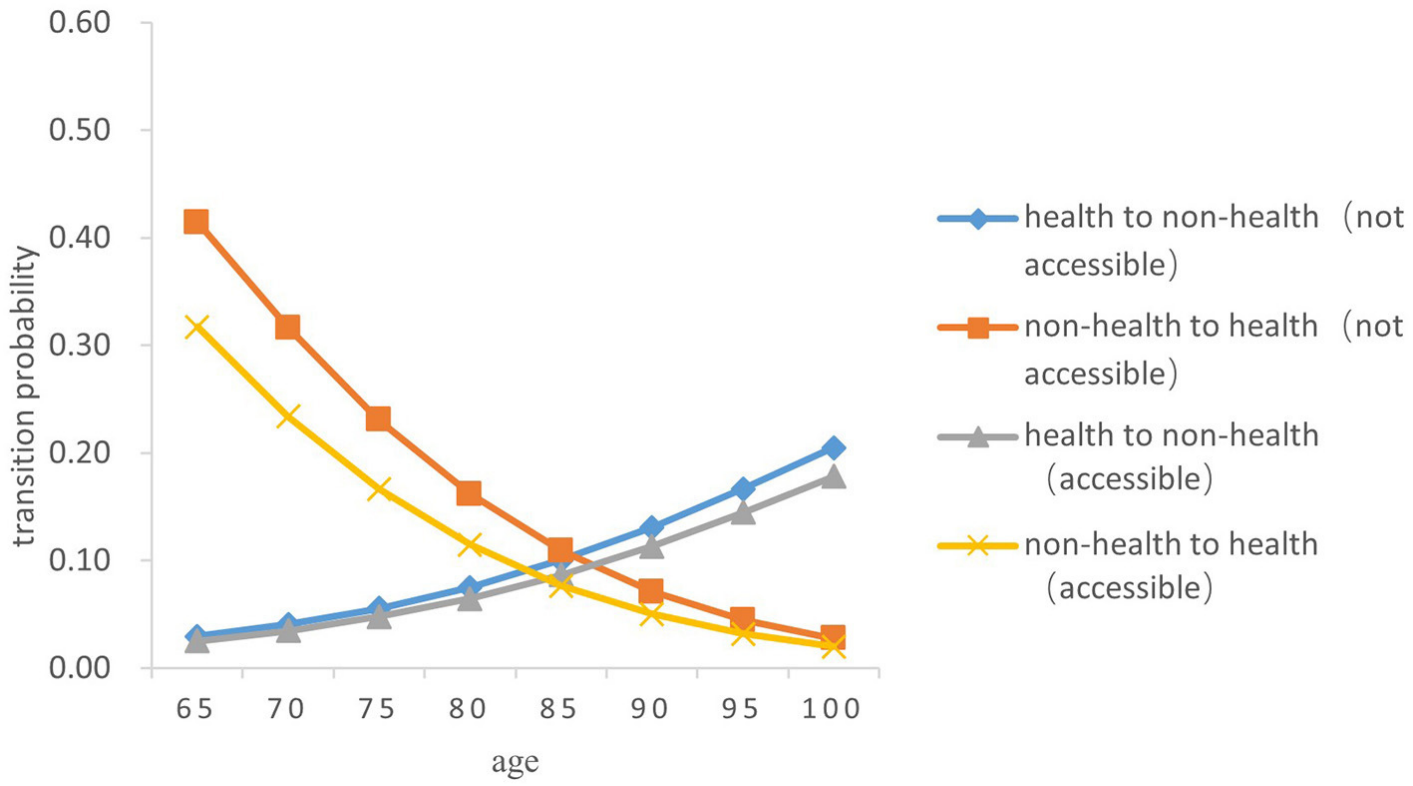
Transition probability curves between health and non-health of urban older female.

**Figure 6:**
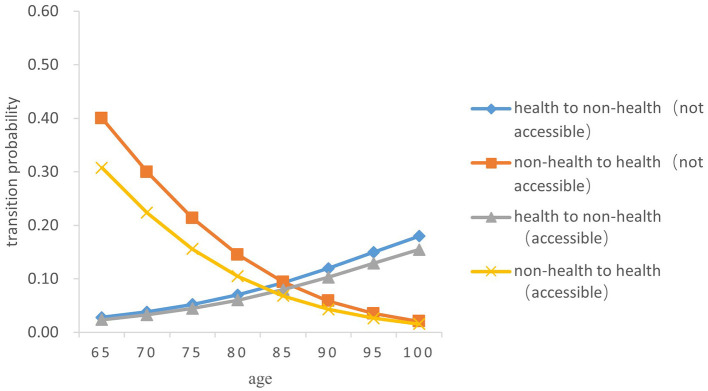
Transition probability curves between health and non-health of urban older male.

Overall, the probability of the transition from health to non-health among older people gradually increases with age, while the probability of the transition risk from non-health to health decreases. In terms of gender, the probability of the transitions between health and non-health in older women was both higher than those in men of the same age (*Z* = −5.238, *P* < 0.05) (As Figures 3–6).

In both urban and rural areas, the probability of the transition from health to non-health of older adults who can receive medical and health services in time during childhood is lower than that of those who fail to receive medical services in time in childhood (*Z* = −5.833, *P* < 0.05). As age increases, the difference between the two transition probability curves from health to non-health gradually expands; However, the probability of the transition from non-health to health in older adults who are not accessible to health services in childhood is higher than that of those who have access to health services in childhood (*Z* = −5.334, *P* < 0.05), and the difference between the two gradually narrows with age.

Specifically, the probability of the transition risk from health to non-health was the lowest among men born in rural areas who were able to receive timely medical and health services during childhood, with a value of 0.022 ± 0.002 at the age 65 and 0.147 ± 0.015 at age 100. Women born in urban areas who did not receive timely health care during childhood had the highest probability of transition risk from health to non-health, with 0.030 ± 0.003 at the age of 65 and 0.205 ± 0.022 at age 100.

### 3.5. Death transition probability

**Figure 7:**
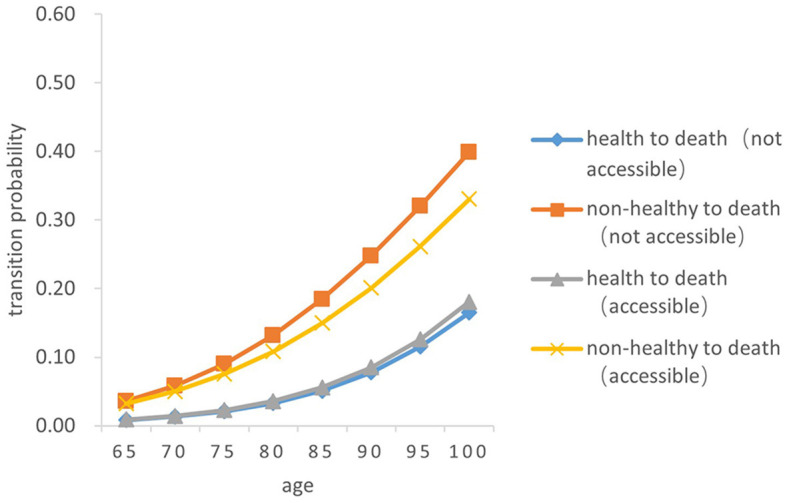
Death transition probability curves of rural older female.

**Figure 8:**
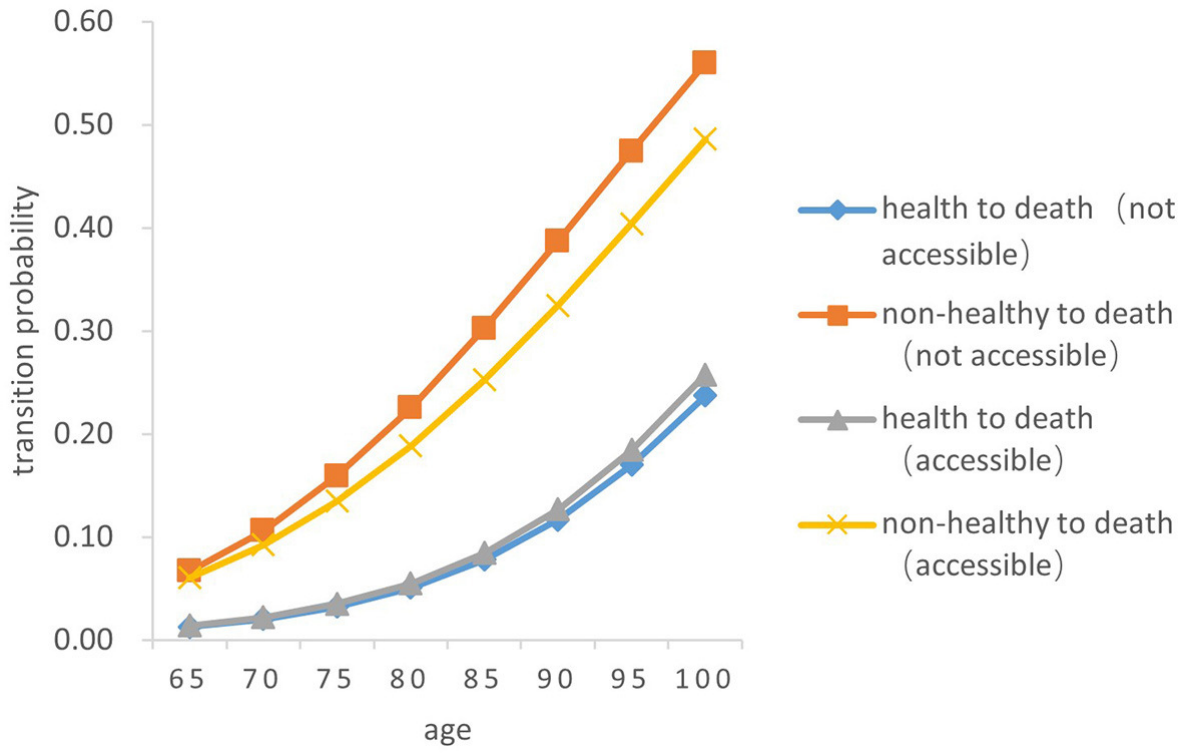
Death transition probability curves of rural older male.

**Figure 9:**
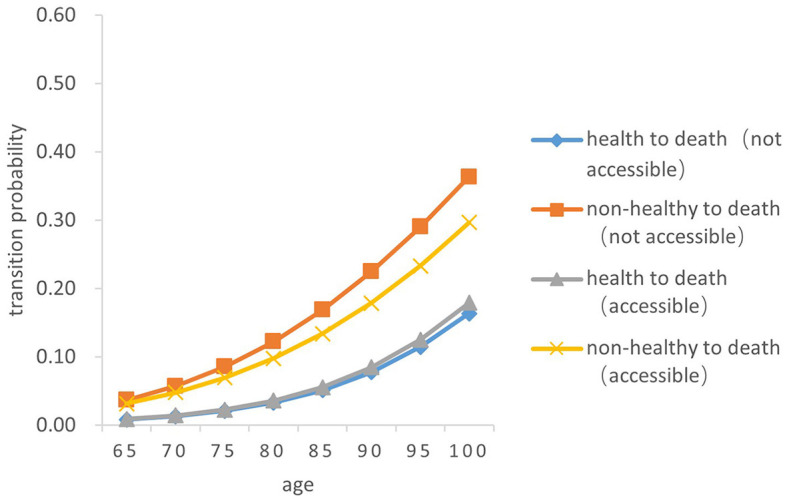
Death transition probability curves of urban older female.

**Figure 10:**
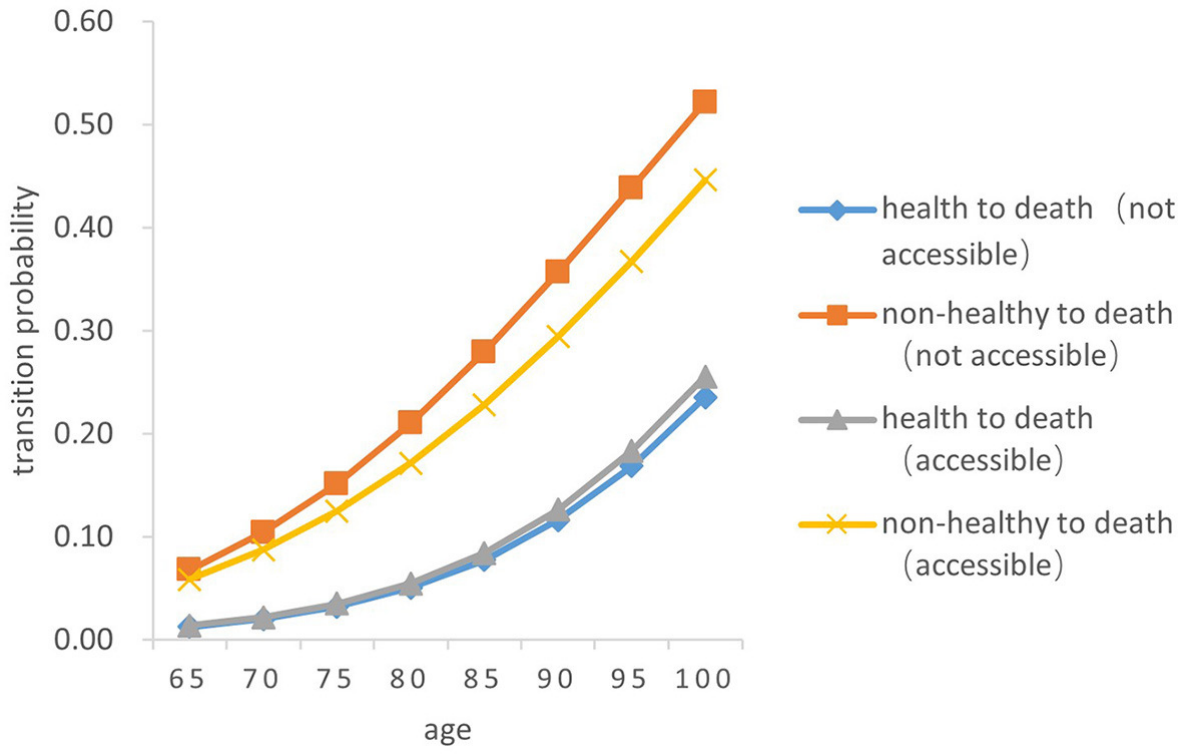
Death transition probability curves of urban older male.

There was a significant difference in the probability of the two transitions between health and death in older adults. Overall, the probability of both transition types (from health to death and from death to health) increased with age, and the probability of the transition from non-health to death increased by a greater magnitude than that from health to death (*Z* = −5.256, *P* < 0.05). From a gender perspective, the transition probability both from health to death and from non-health to death in older women is lower than that in men of the same age (*Z* = −4.748, *P* < 0.05). The probability of the transition risk both from non-health to death and from health to death among older people born in rural areas is higher than that in urban areas (*Z* = −4.529, *P* < 0.05). In both urban and rural areas, the probability of the transition risk from non-health to death among those who failed to receive timely medical and health services in childhood was higher than that of those who received (*Z* = −5.38, *P* < 0.05). The probability of the transition from health to death in those who did not receive timely medical and health services in childhood was slightly higher than that of those who had access to medical and health services in childhood (*Z* = −5.88, *P* < 0.05) (As Figures 7–10).

Specifically, the probability of the transition from health to death was the highest among men born in urban areas and received timely medical and health services during childhood, with 0.01386 ± 0.001 at age 65 and 0.25550 ± 0.256 at age 100. Older women born in rural areas who did not have access to health services during childhood had the lowest transition probability from health to death, with 0.00827 ± 0.001at 65 years old and 0.16510 ± 0.165 at age 100. Older women born in urban areas with timely access to health care during childhood had the lowest transition probability from non-health to death, with 0.03164 ± 0.005 at the age of 65 and 0.29640 ± 0.032 at age 100; Older men born in urban areas who did not receive adequate health care during childhood had the highest transition probability from non-health to death, with 0.06796 ± 0.010 at the age of 65 and 0.52190 ± 0.455 at age 100.

In summary, older people who do not receive timely health care services in childhood have a higher transition risk probability from health to non-health and from non-health to death. The probability of death for older people born in rural areas is lower than that in urban areas. Women have a higher probability of the transitions between health and non-health than those in men, resulting in a lower probability of death.

### 3.6. Healthy life expectancy

As shown in Tables 3, 4, the life expectancy, healthy life expectancy and the proportion of healthy life expectancy in the remaining life expectancy of older adults gradually decreased with age, and there were significant differences with regard to access to health services in childhood, urban or rural areas, and gender. By gender, the healthy life expectancy of older female is higher than that of men (*Z* = −2.38, *P* < 0.05), while its proportion in the remaining life expectancy is lower than that of men (*Z* = −2.74, *P* < 0.05). Urban-rural split, urban-born older adults have a lower healthy life expectancy and a lower proportion of their remaining life expectancy than rural older adults (*Z* = −2.53, *P* < 0.05; *Z* = −4.27, *P* < 0.05). The healthy life expectancy and its proportion in the remaining life expectancy of older adults who received timely medical and health services in childhood are significantly higher than those of their peers (*Z* = −2.19, *P* < 0.05), and the difference is more obvious among rural older female population. Taking those aged 65 as an example, the healthy life expectancy of the older female born in rural areas who can receive childhood medical and health services and those who do not receive them are 17.08 ± 0.21 years and 16.87 ± 0.26 years, respectively, with a difference of 0.21 years. However, there is a difference of 0.82 percentage points under the same condition for the proportion of healthy life expectancy in the remaining life expectancy, which is greater than that of men of the same age and older female born in urban areas at the same time.

**Table 3:** Average life expectancy and healthy life expectancy of rural older population under different childhood medical services.

	**Accessible**			**Not accessible**		
**Female**						
Age	LE	HEL	HLE/LE (%)	LE	HLE	HLE/LE (%)
65	20.02 ± 0.29	17.08 ± 0.21	85.34	19.95 ± 0.23	16.87 ± 0.26	84.52
70	16.13 ± 0.26	13.24 ± 0.18	82.08	16.01 ± 0.20	13.07 ± 0.23	81.58
75	12.65 ± 0.24	9.84 ± 0.17	77.77	12.47 ± 0.19	9.70 ± 0.21	77.78
80	9.64 ± 0.22	6.97 ± 0.15	72.26	9.41 ± 0.17	6.86 ± 0.19	72.88
85	7.18 ± 0.20	4.70 ± 0.14	65.55	6.93 ± 0.16	4.61 ± 0.17	66.60
90	5.26 ± 0.18	3.04 ± 0.13	57.85	5.02 ± 0.14	2.95 ± 0.15	58.77
95	3.85 ± 0.16	1.91 ± 0.12	49.47	3.63 ± 0.12	1.80 ± 0.14	49.45
100	2.86 ± 0.14	1.17 ± 0.12	40.72	2.68 ± 0.11	1.04 ± 0.14	38.95
**Male**						
65	16.67 ± 0.22	14.87 ± 0.20	89.23	16.59 ± 0.25	14.68 ± 0.23	88.49
70	13.06 ± 0.18	11.35 ± 0.17	86.88	13.04 ± 0.21	11.20 ± 0.20	85.90
75	9.92 ± 0.16	8.32 ± 0.15	83.83	9.96 ± 0.19	8.22 ± 0.17	82.53
80	7.33 ± 0.14	5.87 ± 0.13	79.98	7.41 ± 0.16	5.79 ± 0.15	78.24
85	5.32 ± 0.12	4.01 ± 0.12	75.28	5.41 ± 0.14	3.94 ± 0.14	72.96
90	3.85 ± 0.11	2.68 ± 0.12	69.72	3.92 ± 0.13	2.62 ± 0.13	66.66
95	2.81 ± 0.10	1.78 ± 0.12	63.34	2.87 ± 0.10	1.70 ± 0.13	59.31
100	2.11 ± 0.08	1.19 ± 0.12	56.10	2.16 ± 0.08	1.10 ± 0.15	50.88

**Table 4:** Average life expectancy and healthy life expectancy of urban older population under different childhood medical service conditions.

	**Accessible**			**Not accessible**		
**Age**	**LE**	**HEL**	**HLE/LE (%)**	**LE**	**HEL**	**HLE/LE (%)**
**Female**						
65	19.92 ± 0.47	16.25 ± 0.43	81.58	19.70 ± 0.47	16.05 ± 0.41	81.44
70	16.06 ± 0.43	12.46 ± 0.39	77.57	15.79 ± 0.43	12.29 ± 0.37	77.86
75	12.61 ± 0.39	9.13 ± 0.34	72.39	12.28 ± 0.38	9.00 ± 0.33	73.25
80	9.65 ± 0.34	6.36 ± 0.29	65.92	9.28 ± 0.33	6.25 ± 0.29	67.36
85	7.22 ± 0.30	4.21 ± 0.25	58.27	6.85 ± 0.28	4.11 ± 0.24	59.94
90	5.35 ± 0.27	2.66 ± 0.21	49.68	4.99 ± 0.24	2.54 ± 0.21	50.91
95	3.96 ± 0.23	1.61 ± 0.18	40.61	3.64 ± 0.19	1.48 ± 0.18	40.57
100	2.98 ± 0.21	0.94 ± 0.17	31.46	2.70 ± 0.156	0.80 ± 0.17	29.55
**Male**						
65	16.42 ± 0.41	14.25 ± 0.40	86.81	16.39 ± 0.42	14.09 ± 0.38	85.96
70	12.89 ± 0.36	10.77 ± 0.35	83.56	12.81 ± 0.37	10.65 ± 0.33	83.16
75	9.84 ± 0.31	7.82 ± 0.30	79.42	9.72 ± 0.31	7.73 ± 0.28	79.55
80	7.33 ± 0.26	5.45 ± 0.25	74.36	7.18 ± 0.26	5.38 ± 0.24	74.91
85	5.37 ± 0.21	3.68 ± 0.21	68.52	5.22 ± 0.21	3.61 ± 0.21	69.02
90	3.92 ± 0.17	2.43 ± 0.18	62.02	3.79 ± 0.16	2.34 ± 0.19	61.75
95	2.88 ± 0.13	1.58 ± 0.17	54.96	2.78 ± 0.13	1.48 ± 0.19	53.11
100	2.18 ± 0.11	1.03 ± 0.17	47.20	2.10 ± 0.09	0.91 ± 0.19	43.29

## 4. Discussion

### 4.1. Impact of access to health services in childhood on Senile health

The study found that the probability of the transition risk from health to non-health in the older adults who had no access to medical and health services in childhood was higher than that of those who received timely medical services, and the probability of non-health-healthy recovery was low. As a result, older people who did not receive medical and health services in time during childhood not only had a relatively short healthy life expectancy but also a lower proportion of healthy life expectancy in remaining life expectancy. The utilization of medical services for children mostly relies on their original family. In research, the vast majority of older people had relatively poor living conditions in childhood, while the illness of children from low-income families was often underestimated by parents. Even if the price of medical services was low, the utilization level of medical services for children was not significantly improved (18). Parents fail to increase their understanding of their children's health through medical treatment, making it difficult to promote the use of medical services and improve health (19). In addition, childhood illness and failure to seek medical attention in time are likely to cause backward concepts of health in adulthood and lead to bad medical behaviors, such as “delaying in seeking medical service,” consequently, less autonomy in health promotion among older adults, leading to a lower probability of the transition from non-health to health.

This study suggests that the lack of timely access to medical services in childhood leaves a negative effect on health and quality of life later in life through the cumulative effect of disadvantages compared with the early living environment with high medical accessibility. Even though there is a degree of social and class mobility in adulthood, the increase in socioeconomic status and the improvement of the working and living environment cannot fully offset the damage to health in old age due to the lack of medical services in childhood (20). Due to the heterogeneity in the health of older adults, to reduce health inequalities throughout the life cycle and achieve the strategic goal of “health for all,” it is necessary to take into account and strive to meet the diverse needs of different groups of older adults, to develop and optimize interventions that focus on disease prevention and disability mitigation (21).

### 4.2. Significant differences between urban and rural areas in healthy life expectancy and its proportion in the remaining life expectancy

This study shows that under the same childhood health conditions, the healthy life expectancy and its proportion in remaining life expectancy of the rural older adults are higher than those of the urban older adults. On the one hand, there is a certain survivor bias among the rural older adults, that is, the older adults in the poor living environment are very likely to fail to survive due to natural selection. It is this reverse selection mechanism that makes the average life expectancy and healthy life expectancy of the rural older adults higher than that of the urban older adults (22). The older adult living in rural areas engage in more physical labor than their urban counterparts. This not only increases their social activities but also enhances their affirmation and cognition of self-worth, and help to a certain extent to extend the healthy life expectancy and increase its proportion in remaining life expectancy (23, 24). Moreover, after the founding of People's Republic of China, the government attached great value to rural medical and health work, and the “barefoot doctor” policy greatly solved the contradiction between the backwardness of rural medical and health care and the limited resources of the country. The morbidity and mortality rates of infectious and epidemic diseases have been effectively controlled (25), and a good foundation has been laid for the extension of the average life expectancy and healthy life expectancy of the Chinese rural population.

### 4.3. Greater impact of medical services during childhood on the health level of rural women

This paper found that the difference in healthy life expectancy resulting from the ability to seek timely medical care in childhood is most pronounced among rural female older adults. According to the above findings, among the rural-grown female older population, the healthy life expectancy of those who could receive health care during childhood was 0.21 years higher than those who did not. That is, the health benefits of a favorable health care environment or condition during childhood are more likely to tilt toward rural older female through a certain degree of cumulative effect.

Biologically, women are more likely to switch to multiple chronic diseases (26), whereas health care services, as an important means of transitioning from non-health to health, have a greater impact on women's health than men's. The proportion of healthy life expectancy of female older people in the remaining life has always been at a disadvantage. Hence, the gender gap in quality of life from a life course perspective can be narrowed through measures such as optimizing the allocation of rural health care resources and enriching access to health services throughout the life cycle of female. The health condition of older people is constrained by early accumulation of healthy resources, for which childhood is the rapidest stage throughout the life course (27). Therefore, the formation and implementation of health promotion strategies should emphasize in particular the equalization of access to health services during childhood to effectively protect the health rights of girls.

There are still shortcomings in the study. Firstly, the IMaCH software makes the model calculation more complicated and control variables such as individual or family economic status and social environment cannot be included as much as possible. This may lead to omitted variable bias. Secondly, if childhood illnesses are not treated promptly, some survey participants may die prematurely or even not live to the age of 65, so it is possible that the study underestimated the effect of childhood health services on healthy life expectancy. Lastly, there may be some degree of recall bias in their response to the core independent variable survey question: “When you were a child, could you get treatment when you were sick?” However, some bias can be reduced due to the large sample size of the study. The shortcomings of this paper will be further addressed in subsequent studies.

## 5. Conclusion

The absence of medical care during childhood has a long-term adverse impact on an individual's health and, to some extent, reduces healthy life expectancy in old age. From the perspective of life course, it reduces the health stock of individuals and affects the health and quality of life in old age because of the cumulative effect of disadvantages; additionally, it leads to negative perception on health and medical treatment, which is not conducive to establishing a virtuous cycle between medical services and health. Accordingly, it is crucial to take into account the individual demands of older adults and their own conditions, especially the long-term negative effects of adverse experiences in childhood on individual health, in order to improve health in old age, extend healthy life expectancy and improve the quality of the remaining life. Meanwhile, rural female seniors, as a health-sensitive population with access to childhood healthcare services, should be a priority to ensure equal access to healthcare rights and benefits.

## Data availability statement

Publicly available datasets were analyzed in this study. This data can be found here: The data used in this study is openly available from Peking University Open Research Data Platform: https://opendata.pku.edu.cn/.

## Author contributions

BW and JL conceived and designed the study and provided analytical tools. CL analyzed the data and wrote the paper. XH cleansed the data and consulted the literature. XX and QW jointly reviewed and edited the manuscript. All authors contributed to the article and approved the submitted version.
